# COVID-19 pandemic’s disproportionate impact on childhood bereavement for youth of color: Reflections and recommendations

**DOI:** 10.3389/fped.2023.1063449

**Published:** 2023-03-30

**Authors:** Michaeleen Burns, Laura Landry, David Mills, Nichole Carlson, Jillian M. Blueford, Ayelet Talmi

**Affiliations:** ^1^ Evaluation and Research, Judi’s House/JAG Institute for Grieving Children and Families, Aurora, CO, United States; ^2^ Department of Family Medicine, University of Colorado School of Medicine, Aurora, CO, United States; ^3^ Department of Biostatistics and Informatics, Colorado School of Public Health, Aurora, CO, United States; ^4^ Department of Counseling Psychology, University of Denver, Denver, CO, United States; ^5^ Departments of Psychiatry and Pediatrics, University of Colorado School of Medicine, Aurora, CO, United States

**Keywords:** race, hispanic origin, COVID-19, grief, pandemic, childhood bereavement estimation model (CBEM), childhood bereavement, parent death

## Abstract

The COVID-19 pandemic devastated public welfare worldwide, bringing excess deaths connected to causes such as homicide, substance abuse, and heart disease. In the U.S., these mortality increases disproportionally impacted communities of color and contributed to a rise in bereavement among adults and children. The death of an important person is one of the most frequently reported disruptive childhood experiences. According to 2023 Childhood Bereavement Estimation Model (CBEM) results, one in 14 U.S. children will experience the death of a parent by age 18. The current study analyzes the impact of the pandemic on childhood bereavement due to parent death by comparing CBEM results for 2021 and 2020 to the average of annual results for 2016 through 2019 for combined U.S. Census race and Hispanic origin categories. Analyses demonstrate that more than 700,000 U.S. children were newly bereaved due to a parent's death in 2020 and 2021. 2020 increases were observed for each race and Hispanic origin population, ranging from 14.9% to 72.4% compared to the 2016–2019 annual average. Hispanic Asian Pacific Islander and Hispanic Black youth experienced the largest percentage increases, while non-Hispanic white youth experienced the smallest. The results contribute to the growing evidence documenting longstanding and enduring disparities in critical U.S. health outcomes based on race and Hispanic origin. Recommendations for the scale and focus of efforts to understand and address bereavement in a way that accommodates the rising need for support in diverse populations to help all bereaved children find hope and healing are offered.

## Introduction

For most of 2020, SARS-CoV-2 (COVID-19) assaulted United States (U.S.) economic and cultural practices. By late summer, the virus aggressively pushed its way into the leading causes of mortality, accounting for 1 in 8 deaths in the country (1). Simultaneously other death causes increased (2) as mental health concerns soared (3), and decreased access to health care left many uncertain about best practices in maintaining and addressing physical health. While the long-term repercussions of COVID-19 will be studied for decades, one clear effect is an increase in bereavement.

Early estimates show that for each COVID-19 death, nine close friends and family members are bereaved, including thousands of children under 18 (4). Early in the pandemic, virus-related mortality overwhelmingly impacted older adults aged 70 and over—individuals less likely to have children 0–17. As the pandemic continued, the novel coronavirus mutated, and new variants emerged. Late in 2020 and throughout 2021, despite the availability of vaccines, mortality due to the virus increased, the average age of death attributed to the virus decreased (5), and bereavement rates for children under 18 grew steadily. The pandemic's impacts on bereavement reached well beyond direct mortality due to COVID-19, as increases were noted in other leading causes of death. For example, U.S. mortality due to accidental drug overdose has risen for the past two decades, yet overdose deaths spiked more than 31% in 2020, the first year of the pandemic (6). This trend continued in 2021 when the age-adjusted rate of overdose deaths increased by 14% compared to 2020 (7). Similar findings from 2020 to 2021 demonstrate greater than expected adult mortality in all disease conditions except cancer, with the largest increases resulting from accidents, including injuries, assaults, and homicides (8).

The death of a parent during childhood is an adverse experience that increases risks for future behavioral health (9–12), academic (13), and relational problems (14), as well as suicide (15) and earlier mortality (16). Given the potential for disruptive grief reactions with significant short- and long-term consequences, the American Psychiatric Association included Prolonged Grief Disorder in a 2022 text revision of the Diagnostic and Statistical Manual 5, including developmental considerations for children and adolescents (17). The concept of grief as a “disorder” is widely debated in the childhood bereavement field. Estimates show that 10% (18) to 18% (19) of bereaved youth meet the criteria for a diagnosis. Most bereaved children look to their caregivers, peers, and community for support to navigate loss. A recent systematic literature review reflects positive outcomes for bereaved children following participation in preventative interventions aimed at promoting healthy adaptation to the death of a parent (20). Despite this evidence, there is limited access to preventive interventions that have the potential to reduce risk and promote well-being in bereaved youth and families (21). These resources are even more limited in communities with scarce financial, social, and health safety nets.

Despite the clear evidence that childhood bereavement can lead to negative outcomes, efforts to estimate childhood bereavement prevalence rates have been hampered by methodological, reporting, and data source limitations. In the absence of national bereavement tracking systems in the U.S., a statistical estimation model was developed to quantify this public health issue. The Childhood Bereavement Estimation Model (CBEM) is a theory-based probability model incorporating life-table modeling techniques. Burns et al. (22) describe how the CBEM evaluates an input dataset of age-based population, mortality, and natality data for distinct geographies to generate estimates. The CBEM uses publicly available data and incorporates a series of assumptions to derive bereavement estimates. Model results demonstrate fluctuations in childhood bereavement prevalence related to geographic location, age, population density, cause of death, and race and ethnicity. The CBEM offers social service, community support, and health professionals a tool for raising awareness about the magnitude of childhood bereavement and assessing the need for grief services within specific localities. CBEM results ultimately play a role in equipping communities to develop effective preventive bereavement interventions for children that are inclusive and accessible.

Pre-pandemic CBEM results demonstrated that Black and Indigenous children comprise a disproportionate share of bereaved youth in the U.S. (23). For example, Black youth accounted for 16.7% of the U.S. population under age 18, yet they made up 23.9% of bereaved children, a 43% overrepresentation. Analyzing CDC provisional death data from February 2020 to January 2021, Kidman and colleagues (24) estimated a 17.5%–20.2% increase in childhood bereavement due to parent death absent COVID-19. In a study aimed at quantifying COVID-19-associated orphanhood, Hillis et al. (25) demonstrated that the likelihood of experiencing parent or grandparent death due to COVID-19 was up to 4.5 times higher for children of color when compared to non-Hispanic white peers.

The current study analyzed annual childhood bereavement rates from the death of a parent from 2016 to 2021 using the CBEM and compared results from the 2 years of the pandemic (2020 and 2021) to the preceding 4 years (2016–2019) for all U.S. children 0 to 17. Further, the analyses explored the differential toll of bereavement on children based on race and Hispanic origin. Results address increases in bereavement, directly and indirectly, connected to COVID-19. Recommendations for prevention and intervention efforts are offered.

## Materials and methods

Since the initial CBEM publication (22), the model has been updated and refined to increase accuracy. An online CBEM technical appendix documents changes over time that includes quantitative techniques, information sources, and processing approaches (26). Table 1 details the changes relevant to the current analyses examining bereavement in childhood due to parent death.

**Table 1:** Review of CBEM's parental bereavement elements and updates.

CBEM element	Burns et al. 2020	CBEM as of 2023	Relevance to current results
Mortality rate and population data	Mortality and population data is based on 5-year age group values available from CDC WONDER	Mortality and population data is based on 1-year age group values available from CDC WONDER	Shift to 1-year age group data improves the precision of estimates.
Average parent's ages	Fixed values for all geographies and populations	Values calculated specifically according to population and geography	The average age of parent values are calculated specifically for each race and Hispanic origin category
Use of 5-year aggregated data	Mortality rate, deaths, and population data from the most recent 5-year period available from CDC aggregated to the annual average for analyses	Data analyzed as single-year snapshot estimates	Single-year snapshot analyses enable the examination of trends and differences across time

In addition to the assumptions outlined by Burns et al. (22), the current analyses assume a child and their parents all identify with the same race and Hispanic origin. More than 85% of the 5.5 million first live births for the years 2016–2020, with details for both the mother's and father's race and Hispanic origin were born to a mother and father of the same race and Hispanic origin categories (27). While this assumption is restrictive, it is generally representative of most children born in the U.S.

### CBEM input data

All CBEM input data came from the Center for Disease Control and Prevention's Wide-ranging Online Data for Epidemiologic Research (CDC WONDER databases; 28). Data were extracted for each year from 2016 to 2021.

All input variable data were extracted by race and Hispanic origin. Race categories for data from 2016 to 2020 included All Persons (All), American Indian or Alaska Native (AIAN), Asian or Pacific Islander (API), Black or African American (Black), and white. Hispanic origin categories included Hispanic or Latino (Hispanic) and not Hispanic or Latino (non-Hispanic). Given changes in the 2020 U.S. Census, race categories for 2021 also included More than One Race. Data were extracted by population subgroups defined by the interaction of the race and Hispanic origin categories.

Data on the size of the U.S. resident population and the number of U.S. resident deaths by single year of age for those aged 0 through 70 were extracted from CDC WONDER's *Underlying Cause of Death* database (29, 30). The upper limit was chosen as individuals over 70 are less likely to have children 0–17. CDC WONDER's basic natality database (31) and expanded natality database (27) were used to retrieve data on a mother's average age at first live birth.

### Calculated CBEM input variables

CDC WONDER's expanded natality database (27) was used to calculate parents' age at first birth.

*Father's age at first birth*. The mother's average age at first live birth and the total number of births for each race and Hispanic origin category were retrieved within specified father's age group categories. Supplementary Table S1 illustrates the steps used to calculate Father's age at first live birth when data indicated the father and mother have the same race and Hispanic origin.

Supplementary Table S2 provides an example of these calculations showing how the difference in the average age of Black, non-Hispanic fathers and mothers was calculated using 2016 data. Supplementary Table S3 summarizes the mother's and father's age at first birth values incorporated in the “snapshot” analyses. The father's age at first birth variable is the only CBEM input that required calculation outside the model.

*Mother's age (maFB)*. The average age of mothers given a child's age was calculated by adding the mother's age at first live birth to the child's age.

*Father's age (daFB)*. The average age of fathers given the child's age is calculated by adding the calculated father's age at first live birth to the child's age.

*Probability of death (ProbDeathCalc)*. The average annual per person probability of death is calculated by dividing the total number of deaths by the population for a given subgrouping. Supplementary Table S4 compares the crude mortality rates for adults ages 23 to 57 in each subgrouping from 2016 to 2021.

### CBEM snapshot analyses

The 1-year snapshot estimates of children newly bereaved by the death of a parent are a direct reflection of the probability of a parent dying in a year. The estimated number of children of a given age newly bereaved by a parent death (Bcare_ca_) is the product of the number of children of a given age in a year (N_ca_) and the probability of either parent dying in a given year [PDcare_(p1p2)_].

$$\mathrm{Bcar}e_{\mathrm{ca}}=N_{\mathrm{ca}}\;*\;\mathrm{PDcar}e_{\left(p1p2\right)}$$

Assuming parents' deaths can be treated as statistically independent events, the probability of at least one of a child's parents dying [PDcare_(p1p2)_] is the sum of the independent probability of each parent's death (Pcare1_pa_ and Pcare2_pa_) given a child's age, minus the product of these probabilities. In this equation, the parent's probability of death is determined by their age with one parent based on derived mother's and father's age.

$$\mathrm{PDcar}e_{\left(p1p2\right)}=\mathrm{Pcare}1_{\mathrm{pa}}+\mathrm{Pcare}2_{\mathrm{pa}}-(\mathrm{Pcare}1_{\mathrm{pa}}\;*\;\mathrm{Pcare}2_{\mathrm{pa}})$$

## Results

**Figure 1:**
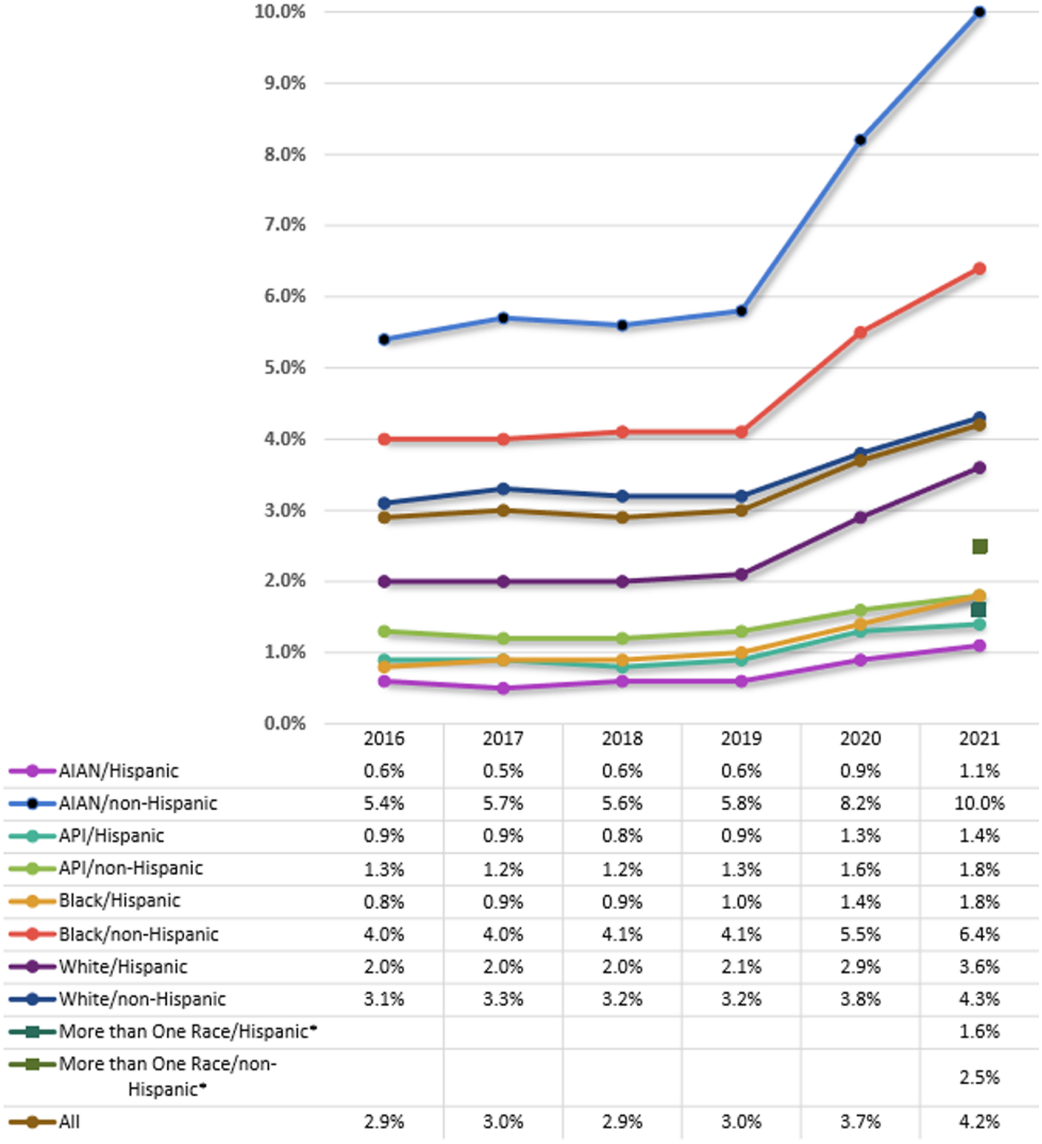
Percent of population under 18 newly bereaved due to parent death. *The more than one race category needs to be included for 2021 to provide a complete summary of the race by hispanic origin population subgroups addressed in the CDC WONDER mortality database.

Figure 1 reflects the disproportionate impact of pandemic bereavement on children of color by displaying the single-year snapshot results for the percentage of the total population of children under 18 in each population category who were newly bereaved in the years 2016–2021. All categories saw increased bereavement in the first and second years of the pandemic. The 2020 U.S. Census allowed respondents to choose more than one race category. Thus, childhood bereavement results for this population are only reported in 2021. In line with previous CBEM results, non-Hispanic American Indian Alaska Native, and non-Hispanic Black youth have the highest bereavement rates across years with 10.0% and 6.4% of the total population of youth in these categories, respectively. Likewise, these two groups experienced the steepest increases in the first 2 years of the pandemic.

Table 2 presents the 1-year snapshot results for newly bereaved children by population group for 2020 with more than 65,000 additional children newly bereaved compared to the annual average from 2016 to 2019, a 24.9% increase. The probability of observing 2020 counts assuming the 2016–2019 distribution is small (<5%) for all race and Hispanic origin groups. Although non-Hispanic white children saw the largest increase in newly bereaved children in 2020 (22,973), the findings are more nuanced. Non-Hispanic white youth experienced the smallest percentage increase in bereavement in 2020 (14.9%) relative to the 2016–2019 annual average. In contrast, Hispanic Asian Pacific Islander (72.0%) and Hispanic Black (72.4%) youth experienced the largest percentage increases. Table 2 also shows that these percentage increases are larger for Hispanic youth across all race groups. Given changes in the 2020 U.S. Census categorization of race, 2021 CBEM results were not analyzed compared to 2016–2020 data. Table 3 has the complete results for the number of newly bereaved children by race and Hispanic origin group for each year from 2016 to 2021.

**Table 2:** Comparisons of CBEM parent death 2020 results with 2016–2019 average by population subgroup.

Population		Number of children newly bereaved		2020 vs. 2016–2019 comparison		Prob ≥ 2020^†^
		2016–2019 annual average (standard deviation)	2020 estimate	Additional children bereaved	Percent increase	
All		262,325 (2,717)	327,643	65,325	24.9%	0.037*
Hispanic or latino	AIAN	407 (52)	673	267	65.6%	0.050*
	API	366 (18)	630	264	72.0%	0.038*
	Black	1,088 (116)	1,875	787	72.4%	0.045*
	White	38,816 (1,890)	58,286	19,470	50.2%	0.040*
Non-hispanic or latino	AIAN	4,954 (151)	7,279	2,326	46.9%	0.038*
	API	7,315 (238)	9,856	2,541	34.7%	0.040*
	Black	55,100 (1,134)	74,218	19,118	34.7%	0.038*
	White	154,410 (2,786)	177,382	22,973	14.9%	0.041*

^†^ Probability of observing 2020 counts, or higher, assuming a distribution of counts with a mean and standard deviation from years 2016–2019. Values were square root transformed to adhere to a normal distribution and the *z*-score used to compute the upper tail probability.

* *p *< .05.

**Table 3:** CBEM parent death 1-year snapshot results 2016–2021 by child’s age and population subgroup.

Year	Population group	Number of children newly bereaved by the death of a parent
2016	All	258,755
	AIAN Hispanic	380
	AIAN non-Hispanic	4,778
	API Hispanic	359
	API non-Hispanic	7,148
	Black Hispanic	971
	Black non-Hispanic	53,640
	White Hispanic	36,951
	White non-Hispanic	152,133
2017	All	264,763
	AIAN Hispanic	346
	AIAN non-Hispanic	5,002
	API Hispanic	355
	API non-Hispanic	7,204
	Black Hispanic	1,033
	Black non-Hispanic	55,057
	White Hispanic	38,100
	White non-Hispanic	158,392
2018	All	261,704
	AIAN Hispanic	446
	AIAN non-Hispanic	4,901
	API Hispanic	358
	API non-Hispanic	7,239
	Black Hispanic	1,106
	Black non-Hispanic	55,306
	White Hispanic	38,805
	White non-Hispanic	154,187
2019	All	264,077
	AIAN Hispanic	454
	AIAN non-Hispanic	5,133
	API Hispanic	393
	API non-Hispanic	7,668
	Black Hispanic	1,241
	Black non-Hispanic	56,398
	White Hispanic	41,409
	White non-Hispanic	152,926
2020	All	327,643
	AIAN Hispanic	673
	AIAN non-Hispanic	7,279
	API Hispanic	630
	API non-Hispanic	9,856
	Black Hispanic	1,875
	Black non-Hispanic	74,218
	White Hispanic	58,286
	White non-Hispanic	177,382
2021	All	383,862
	AIAN Hispanic	782
	AIAN non-Hispanic	7,692
	API Hispanic	512
	API non-Hispanic	9,924
	Black Hispanic	2,118
	Black non-Hispanic	81,228
	White Hispanic	71,401
	White non-Hispanic	198,342
	More than one race Hispanic	1,536
	More than one race non-Hispanic	10,414

## Discussion

In a new application of the CBEM, results quantify one aspect of the COVID-19 pandemic's devastating impact on the U.S. Comparisons of CBEM results from 2020 to 2021 to results from the previous 4 years (2016–2019) clearly highlight the toll of the pandemic on childhood bereavement while also stressing the significance of the issue in the preceding years. Results demonstrate how the pandemic's initial bereavement impacts compounded existing rates of annual bereavement that averaged more than 260,000 children under age 18 being newly bereaved from the death of a parent each year. This number increased by nearly 50%, jumping to more than 383,000 by the end of 2021. The increased childhood bereavement rates were not only attributed to climbing mortality directly connected to COVID-19, but also to indirect impacts of pandemic stress and strain that resulted in rising mortality due to a range of causes such as overdose and homicide death rates. Indeed in 2020, one in five children bereaved by the death of a parent experienced an overdose loss (32). These stigmatized losses may result in increased isolation, guilt, and uncertainty for grieving families.

The nuances revealed by the current analyses show disproportionate increases in bereavement in communities of color. For example, in the first year of the pandemic, compared to the previous 4-year average, rates of childhood bereavement increased by 50% percent or more for all children identified as Hispanic or Latino, regardless of their race, yet the largest jumps were among Hispanic or Latino youth who also identified as Indigenous, Black, and Asian. As the pandemic stretched into 2021, rates of childhood bereavement continued to soar, particularly among Indigenous and Black youth. These analyses show a grim reality where roughly one in ten non-Hispanic Indigenous children under 18 experienced the death of a parent in 2021. Although the current results do not provide data on the cause of these startling differences, the cultural climate of systemic racism and historic oppression are possible contributors. For example, from the start of the pandemic, xenophobic suppositions regarding the origins of COVID-19 fueled anti-Asian sentiment nationwide that contributed to rising rates of depression and anxiety in this community (33). Systemic injustice and lack of access to quality health care contribute to stark inequities that likely resulted in disproportionate death rates for preventable death causes such as heart disease, liver disease, and homicide in communities of color (23).

The current study has important data and methodological differences from other studies of COVID-attributable bereavement or orphanhood in the U.S. (24, 25, 34). First, this study incorporates final, validated CDC WONDER data instead of preliminary estimates. Second, the CBEM results presented here are specific to children grieving the death of a parent. In contrast, prior studies investigated family characteristics or used dynamic family/community models considering bereavement for children raised by non-parent caregivers. Third, the current study's exploration of population subgroups considering race and Hispanic origin provides novel results emphasizing the context of the pandemic's impact on childhood bereavement and aiding resource allocation to provide support. The race and Hispanic origin results demonstrate how the pandemic exacerbated longstanding discrepancies in the magnitude of childhood bereavement for non-white youth.

### Limitations

Although the current study considers all race and Hispanic origin combinations possible with publicly available data, options for population subgroups were constrained by the databases' content. Further, the analyses assume the child and their parents share the same race and Hispanic origin—an assumption that holds for 85% of the population. Although the availability of additional race and ethnicity options within WONDER's databases allows for CBEM expansion, changes in these subgroupings currently limit the potential scope for year-over-year comparisons. Despite using gender-based information to identify relevant annual probabilities of death for each parent, the analyses do not assume specific genders for the child's parents (i.e., the probabilities of death are not gender specific). Finally, CBEM results reflect an undercount of the childhood bereavement phenomenon as it does not include youth bereaved following the death of a parent-figure other than a mother and father. For example, the current results do not include youth bereaved by the death of a custodial grandparent, a situation more common for Black and Indigenous families (35).

### Conclusion

There is a history of research documenting the potential for short- and long-term adverse effects from unaddressed childhood grief (36). The overwhelming increase in mortality created in the wake of the COVID-19 pandemic brought unprecedented attention to the longstanding issue. Without a national database tracking childhood bereavement in the U.S., the CBEM provides a sound approach to estimating prevalence. Disparities in mortality rates across race and Hispanic origin groups in the U.S. result in disproportionate youth bereavement prevalence (37). Before the pandemic, CBEM results illustrated the overrepresentation of children of color among the bereaved in the U.S. The current analyses reflect how the pandemic deepened these gaps and magnified the importance of and the need for timely and effective programming to support bereaved children and families nationwide, whether they are grieving a death due to COVID-19 or any other cause.

The pandemic acutely raised awareness and recognition of bereavement as a critical public health issue, but there is more work to be done in bringing forth culturally relevant, wide-scale, societal understanding and sensitivity. For the estimated 700,000 plus children under the age of 18 who experienced the death of a parent in 2020 and 2021, securing appropriate support may prove challenging. Although local, regional, and national organizations focused on supporting the bereaved youths exists, there are significant gaps across the country, and resources to support these efforts are scarce. Programs like the *Childhood Bereavement Changemaker* initiative (38) acknowledge the field's grassroots origins while bolstering the capacity to make data-informed decisions and enhance impact, but aid for this type of programming is limited. Given the longstanding nature of the issue alongside the recent increases brought on by COVID-19, the following recommendations are offered:

• Broaden public awareness and advocacy efforts that promote grief education and ensure those coping with a death loss are met with sensitivity and responsiveness.
• Assess the size and scope of the issue by creating a national system for quantifying childhood bereavement with mechanisms for annual reporting and benchmarking.
• Develop ongoing processes for screening grief reactions and offering requisite support across systems and institutions (e.g., healthcare, schools).
• Promote research that contributes to understanding the diverse needs of grieving children and translates into practical strategies and efficacious solutions in community settings where bereaved children and families live.
• Strengthen workforce and community capacity to address bereavement by developing and providing appropriate training, tools, and resources. Embed grief-informed knowledge throughout societal systems including education, health care, criminal justice, finance, and government.
• Provide and sustain an affordable continuum of comprehensive prevention and intervention services that address the broad spectrum of bereaved youth. Services should be universally accessible and culturally relevant with a focus on reducing risk and promoting well-being regardless of the acuity of grief reactions.

For the millions of U.S. children and adults bereaved in childhood, the time has come for a comprehensive approach to the development and implementation of resources, support, and services. Researchers, practitioners, educators, policymakers, and advocates must unite to invest in prevention and create social change that ensures a compassionate response to all grieving children and families nationwide.

## Data Availability

Publicly available datasets were analyzed in this study. This data can be found here: Centers for Disease Control and Prevention. Wide-ranging Online Data for Epidemiologic Research. [Internet]. Washington DC, CDC [Updated 2023 January; Cited 2023 January 30] DC 2023. CDC WONDER. Available at: https://wonder.cdc.gov/.
