# Corrigendum: COVID-19 pandemic's disproportionate impact on childhood bereavement for youth of color: reflections and recommendations

**DOI:** 10.3389/fped.2023.1206107

**Published:** 2023-05-24

**Authors:** Michaeleen Burns, Laura Landry, David Mills, Jillian M. Blueford, Nichole Carlson, Ayelet Talmi

**Affiliations:** ^1^ Evaluation and Research, Judi’s House/JAG Institute for Grieving Children and Families, Aurora, CO, United States; ^2^ Department of Family Medicine, University of Colorado School of Medicine, Aurora, CO, United States; ^3^ Department of Biostatistics and Informatics, Colorado School of Public Health, Aurora, CO, United States; ^4^ Department of Counseling Psychology, University of Denver, Denver, CO, United States; ^5^ Departments of Psychiatry and Pediatrics, University of Colorado School of Medicine, Aurora, CO, United States

**Keywords:** race, hispanic origin, COVID-19, grief, pandemic, childhood bereavement estimation model (CBEM), childhood bereavement, parent death

A Corrigendum on COVID-19 pandemic's disproportionate impact on childhood bereavement for youth of color: reflections and recommendations By Burns M, Landry L, Mills D, Carlson N, Blueford JM, and Talmi A. (2023) Front. Pediatr. 11:1063449. doi: 10.3389/fped.2023.1063449

**Text Correction**

In the published article, there was an error. A parenthetic reference to one of the results is incorrect.

A correction has been made to **Results**, Table 2, Paragraph 1. This sentence previously stated:

“Although non-Hispanic white children saw the largest increase in newly bereaved children in 2020 (42,443), the findings are more nuanced.”

The corrected sentence appears below:

“Although non-Hispanic white children saw the largest increase in newly bereaved children in 2020 (22,973), the findings are more nuanced.”

The authors apologize for this error and state that this does not change the scientific conclusions of the article in any way. The original article has been updated.

