# Supplementary material for: COVID-19 pandemic’s disproportionate impact on childhood bereavement for youth of color: Reflections and recommendations
Source: Front Pediatr. 2023 Mar 30;11:1063449. doi: 10.3389/fped.2023.1063449 (PMC10098329; doi:10.3389/fped.2023.1063449)
Supplement: Supplementary file 1 [file Table1.docx]

**Supplement Materials Table 1**

*Steps for calculating father’s age at first live birth**

| Variable | Calculation |
| --- | --- |
| Father’s average age | Fathers in each reported age group category were assigned an average age. The midpoint was used for defined range categories (e.g., 22 years for the *20–24 years* category). Fathers are assumed to be 15 years old in the *age under 15* category. Fathers are assumed to be 55 years old in the *age 55+* category. |
| Mother’s and father’s weighted average ages | The calculation used the total number of births across the *Age of Father* categories, and the assumed and reported average ages for fathers and mothers, respectively. The average mother’s age was subtracted from the average father’s age to produce the difference in parents ages. |
| Father’s age at first live birth | The difference in parents ages was added to the value for the average age of mothers at their first live birth to calculate the average father’s age at a mother’s first live birth. |

*Original data source CDC WONDER expanded natality database.
