# Supplementary material for: COVID-19 pandemic’s disproportionate impact on childhood bereavement for youth of color: Reflections and recommendations
Source: Front Pediatr. 2023 Mar 30;11:1063449. doi: 10.3389/fped.2023.1063449 (PMC10098329; doi:10.3389/fped.2023.1063449)
Supplement: Supplementary file 2 [file Table2.docx]

**Table 2**

*Comparisons of CBEM parent death 2020 results with 2016-2019 average by population subgroup*

| Population | | Number of children  newly bereaved | |  | | 2020 vs 2016-2019  comparison | | | | Prob ≥ 2020^†^ |
| --- | --- | --- | --- | --- | --- | --- | --- | --- | --- | --- |
|  |  | 2016-2019 annual average (standard deviation) | 2020 Estimate | |  | | Additional  children  bereaved | | Percent increase |  |
| All | | 262,325 (2,717) | 327,643 | |  | | 65,325 | 24.9% | | 0.037* |
| Hispanic or Latino | AIAN | 407 (52) | 673 | |  | | 267 | 65.6% | | 0.050* |
|  | API | 366 (18) | 630 | |  | | 264 | 72.0% | | 0.038* |
|  | Black | 1,088 (116) | 1,875 | |  | | 787 | 72.4% | | 0.045* |
|  | White | 38,816 (1,890) | 58,286 | |  | | 19,470 | 50.2% | | 0.040* |
| Non- Hispanic or Latino | AIAN | 4,954 (151) | 7,279 | |  | | 2,326 | 46.9% | | 0.038* |
|  | API | 7,315 (238) | 9,856 | |  | | 2,541 | 34.7% | | 0.040* |
|  | Black | 55,100 (1,134) | 74,218 | |  | | 19,118 | 34.7% | | 0.038* |
|  | White | 154,410 (2,786) | 177,382 | |  | | 22,973 | 14.9% | | 0.041* |

^†^Probability of observing 2020 counts, or higher, assuming a distribution of counts with a mean and standard deviation from years 2016-2019. Values were square root transformed to adhere to a normal distribution and the z-score used to compute the upper tail probability.

## *p <. 05
