# Supplementary material for: COVID-19 pandemic’s disproportionate impact on childhood bereavement for youth of color: Reflections and recommendations
Source: Front Pediatr. 2023 Mar 30;11:1063449. doi: 10.3389/fped.2023.1063449 (PMC10098329; doi:10.3389/fped.2023.1063449)
Supplement: Supplementary file 3 [file Table3.docx]

**Supplement Materials Table 3**

*Details on rounded average ages for mothers and fathers at a mother’s first live birth by year and population group*

| Mother's race/Hispanic origin | Year | Average age of mother | Average difference in maFB and daFB | Rounded maFB for CBEM | Rounded daFB for CBEM |
| --- | --- | --- | --- | --- | --- |
| All Persons | 2016 | 26.65 | 2.41 | 27.00 | 29.00 |
|  | 2017 | 26.78 | 2.39 | 27.00 | 29.00 |
|  | 2018 | 26.93 | 2.34 | 27.00 | 29.00 |
|  | 2019 | 27.01 | 2.28 | 27.00 | 29.00 |
|  | 2020 | 27.13 | 2.22 | 27.00 | 29.00 |
|  | 2021 | 27.34 | 2.13 | 27.00 | 29.00 |
| American Indian or Alaska Native/Hispanic or Latino | 2016 | 23.19 | 2.32 | 23.00 | 26.00 |
|  | 2017 | 23.56 | 2.88 | 24.00 | 26.00 |
|  | 2018 | 23.62 | 2.51 | 24.00 | 26.00 |
|  | 2019 | 23.77 | 2.32 | 24.00 | 26.00 |
|  | 2020 | 24.07 | 2.78 | 24.00 | 27.00 |
|  | 2021 | 24.19 | 2.67 | 24.00 | 27.00 |
| American Indian or Alaska Native/non-Hispanic or Latino | 2016 | 23.22 | 2.46 | 23.00 | 26.00 |
|  | 2017 | 23.30 | 2.36 | 23.00 | 26.00 |
|  | 2018 | 23.47 | 2.21 | 23.00 | 26.00 |
|  | 2019 | 23.50 | 2.20 | 24.00 | 26.00 |
|  | 2020 | 23.76 | 2.40 | 24.00 | 26.00 |
|  | 2021 | 23.90 | 2.10 | 24.00 | 26.00 |
| Asian or Pacific Islander/Hispanic or Latino | 2016 | 25.09 | 3.12 | 25.00 | 28.00 |
|  | 2017 | 25.19 | 2.63 | 25.00 | 28.00 |
|  | 2018 | 25.88 | 2.61 | 26.00 | 28.00 |
|  | 2019 | 25.86 | 2.64 | 26.00 | 29.00 |
|  | 2020 | 26.09 | 2.65 | 26.00 | 29.00 |
|  | 2021 | 26.46 | 2.64 | 26.00 | 29.00 |
| Asian or Pacific Islander/non-Hispanic or Latino | 2016 | 29.99 | 2.93 | 30.00 | 33.00 |
|  | 2017 | 30.13 | 2.91 | 30.00 | 33.00 |
|  | 2018 | 30.36 | 2.84 | 30.00 | 33.00 |
|  | 2019 | 30.54 | 2.73 | 31.00 | 33.00 |
|  | 2020 | 30.73 | 2.64 | 31.00 | 33.00 |
|  | 2021 | 31.06 | 2.48 | 31.00 | 34.00 |

**Supplement Materials Table 3 (Cont.)**

*Details on rounded average ages for mothers and fathers at a mother’s first live birth by year and population group*

| Mother's race/Hispanic origin | Year | Average age of mother | Average difference in maFB and daFB | Rounded maFB for CBEM | Rounded daFB for CBEM |
| --- | --- | --- | --- | --- | --- |
| Black/Hispanic or Latino | 2016 | 24.34 | 3.05 | 24.00 | 27.00 |
|  | 2017 | 24.40 | 2.74 | 24.00 | 27.00 |
|  | 2018 | 24.51 | 2.91 | 25.00 | 27.00 |
|  | 2019 | 24.58 | 2.68 | 25.00 | 27.00 |
|  | 2020 | 24.76 | 2.52 | 25.00 | 27.00 |
|  | 2021 | 25.00 | 2.55 | 25.00 | 28.00 |
| Black/non-Hispanic or Latino | 2016 | 24.79 | 2.73 | 25.00 | 28.00 |
|  | 2017 | 24.94 | 2.73 | 25.00 | 28.00 |
|  | 2018 | 25.14 | 2.70 | 25.00 | 28.00 |
|  | 2019 | 25.16 | 2.68 | 25.00 | 28.00 |
|  | 2020 | 25.31 | 2.63 | 25.00 | 28.00 |
|  | 2021 | 25.54 | 2.54 | 26.00 | 28.00 |
| White/Hispanic or Latino | 2016 | 24.76 | 2.48 | 25.00 | 27.00 |
|  | 2017 | 24.89 | 2.46 | 25.00 | 27.00 |
|  | 2018 | 25.05 | 2.42 | 25.00 | 27.00 |
|  | 2019 | 25.16 | 2.33 | 25.00 | 27.00 |
|  | 2020 | 25.33 | 2.26 | 25.00 | 28.00 |
|  | 2021 | 25.56 | 2.21 | 26.00 | 28.00 |
| White/non-Hispanic or Latino | 2016 | 27.40 | 2.18 | 27.00 | 30.00 |
|  | 2017 | 27.56 | 2.16 | 28.00 | 30.00 |
|  | 2018 | 27.70 | 2.11 | 28.00 | 30.00 |
|  | 2019 | 27.80 | 2.06 | 28.00 | 30.00 |
|  | 2020 | 27.94 | 1.99 | 28.00 | 30.00 |
|  | 2021 | 28.14 | 1.91 | 28.00 | 30.00 |
| More than One Race/  Hispanic or Latino* | 2021 | 25.58 | 2.00 | 26.00 | 28.00 |
| More than One Race/  non-Hispanic or Latino* | 2021 | 25.95 | 2.24 | 26.00 | 28.00 |

^*^ The *More than One Race* category needs to be included for 2021 to provide a complete summary of the race by Hispanic origin population subgroups addressed in the WONDER mortality database.
