# Supplementary material for: COVID-19 pandemic’s disproportionate impact on childhood bereavement for youth of color: Reflections and recommendations
Source: Front Pediatr. 2023 Mar 30;11:1063449. doi: 10.3389/fped.2023.1063449 (PMC10098329; doi:10.3389/fped.2023.1063449)
Supplement: Supplementary file 4 [file Table4.docx]

**Supplement Materials Table 4**

*Crude mortality rate^†^ for parents* in each category*

| Population |  | 2021 | 2020 | Average 2016-2019 |
| --- | --- | --- | --- | --- |
| **All** |  | **359.2** | **319.8** | **269.0** |
| Hispanic or Latino |  |  |  |  |
|  | AIAN | 73.8 | 56.2 | 42.2 |
|  | API | 106.5 | 94.4 | 68.1 |
|  | Black | 114.1 | 96.8 | 64.5 |
|  | White | 306.2 | 267.5 | 180.1 |
|  | More than One Race | 115.7 | NA | NA |
| Non-Hispanic or Latino |  |  |  |  |
|  | AIAN | 831.6 | 672.2 | 473.1 |
|  | API | 135.1 | 126.2 | 100.8 |
|  | Black | 552.4 | 489.0 | 381.5 |
|  | White | 366.6 | 321.7 | 291.1 |
|  | More than One Race | 181.5 | NA | NA |

^†^Deaths per 100,000 of the population

*Adults 23-57 years old. The ages most likely to have children 0-17 years old.
